# Understanding the Risk Factors and Long-Term Consequences of Cisplatin-Associated Acute Kidney Injury: An Observational Cohort Study

**DOI:** 10.1371/journal.pone.0142225

**Published:** 2015-11-10

**Authors:** Zeenat Yousuf Bhat, Pravit Cadnapaphornchai, Kevin Ginsburg, Milani Sivagnanam, Shamit Chopra, Corey K. Treadway, Ho-Sheng Lin, George Yoo, Ammar Sukari, Mona D. Doshi

**Affiliations:** 1 Wayne State University, School of Medicine, Detroit, Michigan, United States of America; 2 Patel Hospital, Civil Lines, Jalandhar City, Punjab, India; 3 Cass Street, Ear, Nose & Throat Associates, Traverse City, Michigan, United States of America; 4 Karmanos Cancer Institute, Detroit, Michigan, United States of America; National Institutes of Health, UNITED STATES

## Abstract

Acute kidney injury (AKI) is a well-known complication of cisplatin-based chemotherapy; however, its impact on long-term patient survival is unclear. We sought to determine the incidence and risk factors for development of cisplatin-associated AKI and its impact on long-term renal function and patient survival. We identified 233 patients who received 629 cycles of high-dose cisplatin (99±9mg/m^2^) for treatment of head and neck cancer between 2005 and 2011. These subjects were reviewed for development of AKI. Cisplatin nephrotoxicity (CN) was defined as persistent rise in serum creatinine, with a concomitant decline in serum magnesium and potassium, in absence of use of nephrotoxic agents and not reversed with hydration. All patients were hydrated per protocol and none had baseline glomerular filtration rate (GFR) via CKD-EPI<60mL/min/1.73m^2^. The patients were grouped based on development of AKI and were staged for levels of injury, per KDIGO-AKI definition. Renal function was assessed via serum creatinine and estimated glomerular filtration rate (eGFR) via CKD-EPI at baseline, 6- and 12-months. Patients with AKI were screened for the absence of nephrotoxic medication use and a temporal decline in serum potassium and magnesium levels. Logistic regression models were constructed to determine risk factors for cisplatin-associated AKI. Twelve-month renal function was compared among groups using ANOVA. Kaplan-Maier curves and Cox proportional hazard models were constructed to study its impact on patient survival. Of 233 patients, 158(68%) developed AKI; 77 (49%) developed stage I, 55 (35%) developed stage II, and 26 (16%) developed stage III AKI. Their serum potassium and magnesium levels correlated negatively with level of injury (p<0.05). African American race was a significant risk factor for cisplatin-associated AKI, OR 2.8 (95% CI 1.3 to 6.3) and 2.8 (95% CI 1.2 to 6.7) patients with stage III AKI had the lowest eGFR value at 12 months (p = 0.05) and long-term patient survival (HR 2.1; p<0.01) than patients with no or lower grades of AKI. Most common causes of death were recurrent cancer (44%) or secondary malignancy elsewhere (40%). Cisplatin-associated severe AKI occurs in 20% of the patients and has a negative impact on long-term renal function and patient survival. PEG tube placement may be protective and should be considered in high risk-patients.

## Introduction

Cisplatin is an alkylating chemotherapeutic agent widely used in treatment of a variety of cancers. However, its efficacy is limited by significant side effects such as nephrotoxicity, neurotoxicity, and ototoxicity [1]. Renal toxicity occurs in a third of patients and is dose dependent [2]. Prior literature suggests that aggressive hydration during cisplatin infusion along with mannitol may reduce nephrotoxicity by increasing urinary flow rate and decreasing the contact time between the drug and renal tubules [3]. Thus, fluids and mannitol have become standard of care, especially with high dose cisplatin infusion [4]. Cisplatin-associated AKI results in irreversible renal injury [5]. In general population, reduced renal function is associated with inferior patient survival; partially explained by the presence of common risk factors such as diabetes and hypertension which are linked to both kidney and heart disease. Currently, there is only one study from Japan [6] showing inferior patient survival in patients with cisplatin-associated severe AKI. The authors reported that history of diabetes, cardiac disease, and stage IV cancers were significant risk factors for development of severe AKI. It is unclear if the increased mortality is due to cardiac disease versus progression of cancer due to cessation of effective chemotherapy.

The aims of the present study are to determine the incidence and risk factors for development of AKI with use of high dose cisplatin in the current era of judicious patient selection and adequate preconditioning regimen, consisting of fluids and mannitol and also to understand its impact on long-term renal function and patient survival.

## Methods

### Study design & setting

This is a retrospective cohort study of all adult patients receiving cisplatin based chemotherapy for treatment of head and neck cancer between 2005 and 2011 at Karmanos Cancer Center, Detroit, Michigan. Cisplatin-associated AKI is dose dependent, and therefore, we limited our study to include patients receiving highest dose of cisplatin (head and neck cancer patients). The study protocol was approved by Wayne State University Institutional Review Board (approval number 056013MP2E). Patient records/information was anonymized and de-identified prior to analysis.

### Patients

All patients had their baseline renal function assessed via serum creatinine and the oncologist prescribed cisplatin based chemotherapy only for those with serum creatinine <1.5 mg/dL. Patients were admitted to the hospital for an overnight stay and received 25 grams of mannitol and a liter of D5 water/normal saline before and two liters immediately after cisplatin infusion. Additional hydration with a liter of D5 water/normal saline was also provided on days 3 and 7 after chemotherapy. Serum potassium, magnesium, and creatinine were obtained before starting chemotherapy and again on days 1, 3 and 7. Patients received additional cycles at three-week intervals, per the recommendation of the oncologist. The dosing, hydration, and laboratory tests were obtained at the time points described above. Over 90% of these patients also received concomitant radiation therapy.

### Data

Patient charts were reviewed and the following data were collected: demographics, history of hypertension, diabetes, smoking and alcohol; weight, height, body mass index (BMI); site and stage of cancer, dose of cisplatin per cycle and number of cycles; serum creatinine, potassium, and magnesium at baseline, after each cycle, and at 1 and 12 months after last cycle; use of medications that can affect kidney function such as diuretics, angiotensin converting enzyme inhibitors, angiotensin receptor inhibitors, NSAIDS, aminoglycoside, and CT with intravenous contrast prior to every cycle. PEG tube placements for fluid and nutritional support were also noted for every patient. PEG was placed as per the discretion of the oncologist and indication was losing 10% of baseline weight while on chemo-radiation. Renal function was assessed using serum creatinine and eGFR values obtained via CKD-EPI equation [7].

### Outcomes and variables

The primary end point was incidence and risk factors for development of AKI associated with high dose cisplatin infusion. The serum creatinine obtained on four occasions: at days 0 and 1 during their hospital stay for infusion and thereafter on days 3 and 7 at their out-patient visit after infusion. AKI was attributed to cisplatin only if there was a persistent rise in serum creatinine (on both day 3 and day 7 from baseline) with a concomitant decline in serum magnesium and potassium, in absence of use of nephrotoxic agents and not reversed with hydration. Development of AKI was evaluated after each cycle. The grading of severity of renal dysfunction was per KDIGO: Stage I: 1.5 to 1.9 times or ≥ 0.3 mg/dL pre-treatment value; Stage II: 2.0–2.9 times pre-treatment value and Stage III: ≥ 3.0 times pre-treatment value or increase in serum creatinine to ≥ 4.0 mg/dL or requiring renal replacement therapy. Thus, there were four groups of patients: no AKI, Stage I AKI, Stage II AKI, and Stage III AKI.

The secondary outcomes of interest were impact of cisplatin-associated AKI on long-term kidney function and patient survival. Twelve-month eGFR was calculated on all patients and was compared among the four groups of patients. Fraction of patients with GFR < 60ml/min was also compared among the groups. The Surveillance, Epidemiology and End Results (SEER) dataset carries detailed information on all cancer patients including their vital status. The vital status is matched with the National Death Index and Death File from State of Michigan. The dataset is held by the Epidemiology Research Core at Karmanos Cancer Institute and was used to retrieve vital status and cause of death on all patients included in this study.

### Statistical Methods

The incidence of cisplatin-associated AKI was calculated for the entire cohort and also for each stage. Changes in eGFR, magnesium, potassium at baseline and at time of injury were assessed. The baseline characteristics, past medical history, site and stage of cancer, dose and number of cycles of cisplatin, use of PEG tube, serum creatinine, potassium, and magnesium prior to treatment were compared among the four groups. Multivariate logistic regression models were created to assess risk factors for cisplatin-associated AKI. Kaplan-Meier curves were constructed to plot patient survival in the four groups based on severity of AKI and log rank test was used to compare their survival. Cox proportional hazard models were constructed to study the impact of AKI on patient survival after adjusting for differences in baseline demographics and clinical characteristics. The continuous variables were analyzed via ANOVA or Kruskal-Wallis tests, followed by Bonferroni correction for multiple group comparisons. The categorical variables were analyzed via chi-square test or Fisher’s exact test. For these tests, a two sided p-value ≤ 0.05 was considered statistically significant. SAS version 9.3 (SAS Institute, Cary, NC) was used to perform analyses and generate graphics.

## Results

The 233 patients with head and neck cancer received a total of 629 cycles of cisplatin during the study period. One-hundred ninety-two (83%) patients underwent more than one cycle, and the median number of cycles was 3 (range 1–6). The mean dose of cisplatin at each cycle was 99±9 mg/m^2^. The mean age of the study patients was 53.6±9.3 years, 76% were males, and 30% were African American. The mean eGFR for the cohort was 116.7±9.1 mL/min/1.732m^2^.

**Fig 1:**
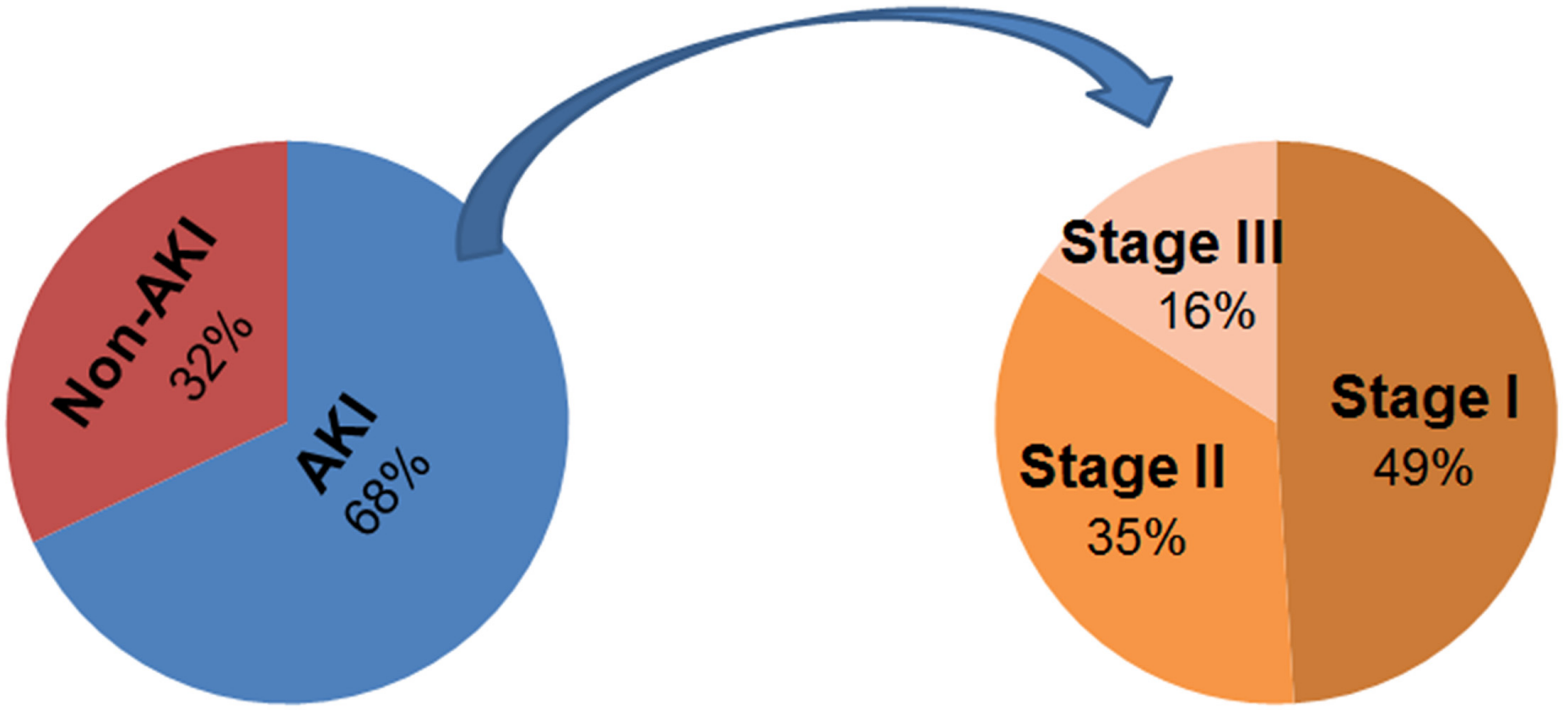
Pie-Chart of AKI and its severity.

Of the 233 patients, 158 (68%) did develop AKI; 77 (49%) developed Stage I, 55 (35%) developed Stage II, and 26 (16%) developed Stage III AKI (Fig 1). None of the patients with Stage III AKI required dialysis. The baseline characteristics of patients in each of the four groups are shown in Table 1. The mean age, gender, history of hypertension, alcohol consumption, height, weight, BMI, cisplatin dose, cancer site and stage, and PEG tube use did not differ among the groups. African Americans, patients with history of diabetes, and smokers were more likely to have higher stages of cisplatin-associated AKI (p<0.05). While the dose per cycle was not different among the four groups, patients with higher Stages of AKI received fewer cycles of cisplatin, and therefore, had lower cumulative dose of cisplatin, possibly due to interruption of therapy due to AKI. The baseline serum creatinine, potassium, and magnesium were statistically significantly lower in patients with higher stages of AKI, possibly reflecting poor nutritional status as suggested by an inverse trend in weight and severity of AKI. Higher stages of cisplatin-associated AKI were associated with a greater decline in serum potassium (Stage II & III vs. 0; p<0.01) and magnesium (Stage II vs. 0; p <0.01).

**Table 1:** Characteristics of patients with and without Acute Kidney Injury.

	No AKI (n = 108, 46%)	AKI (n = 125, 54%)			p-value
		Stage I (n = 41, 33%)	Stage II (n = 58, 47%)	Stage III (n = 26, 20%)	
Age	53.5 ± 9	56.7 ± 10	52.3 ± 10	53.5 ± 9	0.23
Male gender	77%	80%	78%	69%	0.76
% African-American	18	29	41	58	<0.01
History of hypertension	35%	37%	52%	54%	0.08
History of diabetes	6%	0%	14%	4%	0.04
History of smoking	70%	85%	88%	92%	<0.01
History of alcohol	71%	78%	78%	77%	0.72
Weight (kg)	78.6±21.8	78.5±18.8	76.7±21.8	71.8±16.8	0.49
Height (cm)	173.5±8.3	172.5±9.5	173.8±8.5	174.1±7.7	0.85
BMI (kg/m^2^)	26.1±6.1	26.3±5.7	25.5±6.9	23.6±4.9	0.27
Cancer Site					0.66
Lip & Oral Cavity	35%	41%	31%	31%	
Pharynx	48%	41%	50%	61%	
Larynx	8%	0%	5%	0%	
Sinus & Salivary Glands	14%	17%	14%	8%	
Cancer Stage					0.24
I	3%	2%	2%	4%	
II	8%	7%	3%	0%	
III	19%	15%	10%	15%	
IV	70%	76%	85%	81%	
Cisplatin dose (mg/m^2^)	94.2 ± 9	96.8 ± 87	96.4 ± 9	97.6 ± 7	0.57
Patients with ≥ 3 Cycles	65%	51%	57%	36%	0.05
PEG tube placement	46%	41%	40%	54%	0.61
Serum Creatinine (mg/dL)*	0.85 ± 0.2	0.85 ± 0.2	0.77 ± 0.9	0.69 ± 0.9	<0.01
Serum Potassium (mEq/L)	4.2±0.4	4.2±0.5	4.9±0.4	3.9±0.4	0.04
Serum Magnesium (mEq/L)**	1.9±0.2	1.9±0.3	1.8±0.3	1.8±0.3	0.01

*multiply by 88.4 to convert mg/dL to μmol/L

**multiply by 0.5 to convert mEq/L to mmol/L

**Fig 2:**
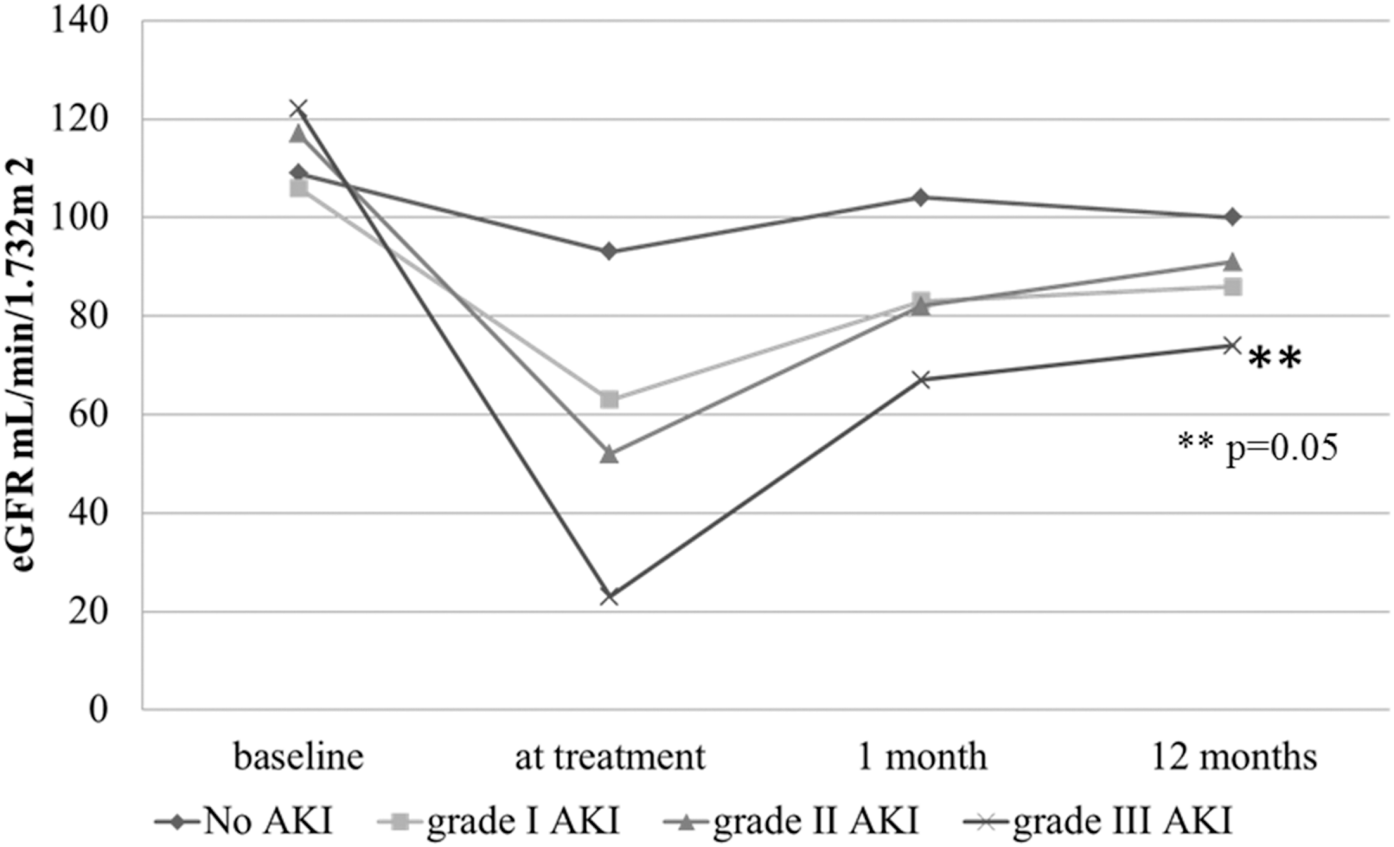
Changes in eGFR at baseline, at treatment/AKI, 1 and 12 months.

Multivariate analyses showed that only African American race is a risk factor for development of CN (odds ratio 2.8, 95% CI 1.3 to 6.3; *p* = 0.01, Table 2). Fig 2 shows the trend in eGFR at baseline, at treatment/AKI and 1 and 12 months thereafter. The use of cisplatin was associated with a decline in eGFR in all patients and the magnitude of decline increased with severity of AKI. The eGFR improved at 1 month but never returned to baseline and remained persistently low at 12 months. Patients with Stage III AKI had the greatest decline in eGFR from baseline to 12 months than patients with no AKI (38% versus 7%; p<0.01) and had the lowest eGFR at 12 months 75±34 mL/min/1.732m^2^; p = 0.05; Fraction of patients with eGFR <60 mL/min increased with severity of AKI (22, 31, 31, 54%; respectively p = 0.02).

**Table 2:** Risk factors for cisplatin nephrotoxicity in multivariate analyses.

Risk Factor	OR (95% Confidence Interval)	p value
Age, years	1.00 (0.97–1.04)	0.97
Male gender (vs. Female)	1.43 (0.66–3.09)	0.37
African American (vs. White)	2.65 (1.25–5.59)	0.01
History of hypertension (Yes vs. No)	1.60 (0.84–3.06)	0.15
History of diabetes	0.74 (0.22–2.46)	0.63
History of smoking	1.75 (0.84–3.62)	0.13

**Fig 3:**
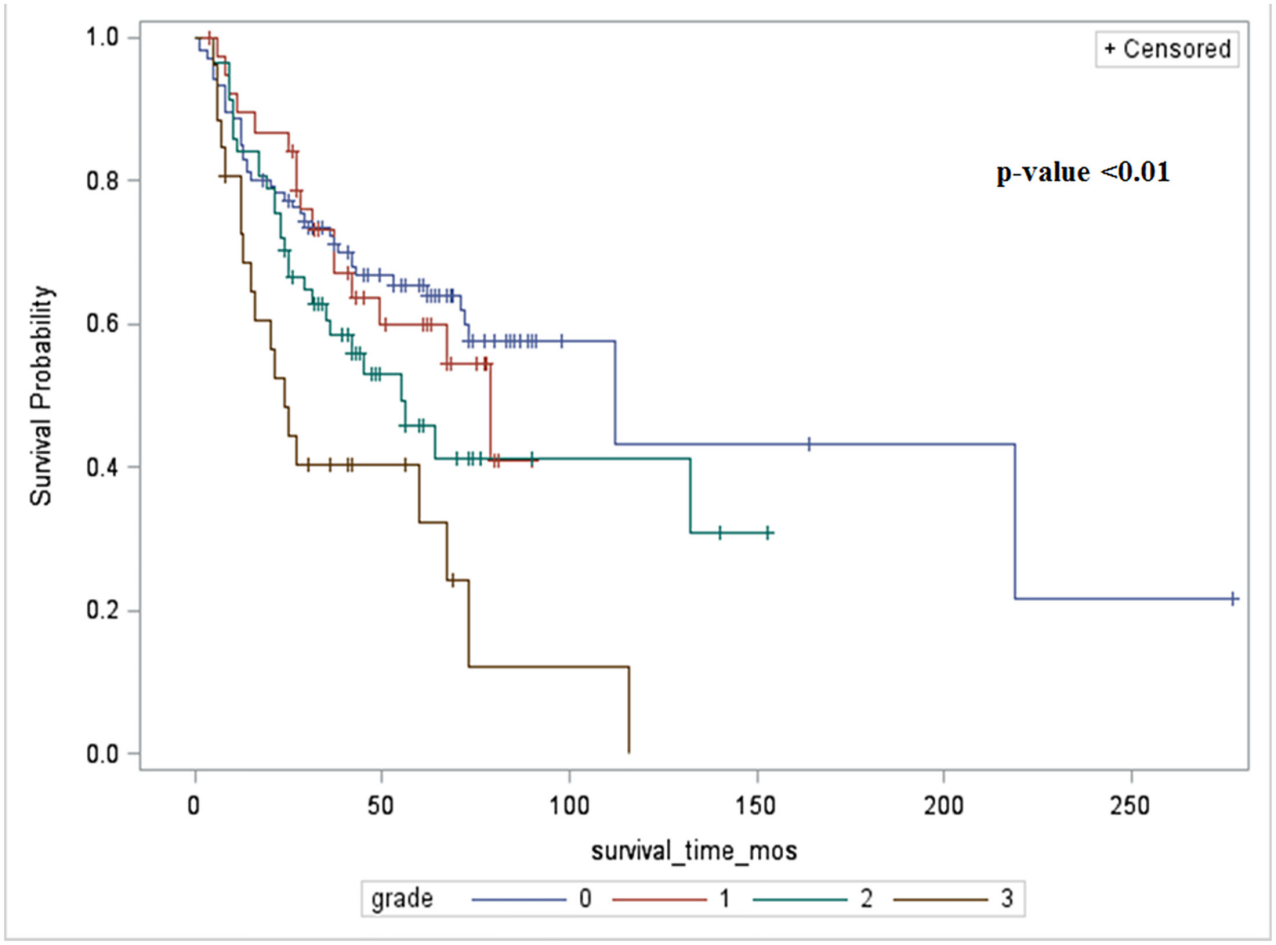
Long term patient survival based on severity of AKI.

Fig 3 shows Kaplan Meier curves for patient survival based on stages of AKI. The median survival among patients with no AKI, Stage I, Stage II and Stage III AKI was 112, 79, 55, 24 months respectively. Patients with Stage III AKI had lower survival than patients that did not develop AKI or developed Stage I or II AKI; p <0.01. On multivariate analyses using Cox proportional hazard model, the patients with Stage III AKI continued to have lower survival with hazard ratio 2.1; p = 0.01. The risk of death was higher among patients with AKI than no AKI [HR (95%CI) are as follows: grade I: 1.54 (0.92–2.61); p = 0.09, grade II: 1.76 (1.02–3.07); p = 0.04 and grade III 3.49 (1.91–6.40); p <0.01]. The causes of death were predominantly related to recurrent cancer (44%) or development of secondary malignancy elsewhere (40%). Only a few were due to cardiovascular or infectious reasons (12%). There were no differences in causes of death by stage of nephrotoxicity (p = 0.69).

## Discussion

Our study reports that over half of the patients receiving high dose cisplatin and radiation for treatment of head and neck cancer develop AKI despite careful patient selection, liberal hydration, and use of mannitol to maintain urinary flow. Unlike prior reports, all cases of AKI were carefully screened for common etiologies of renal dysfunction and the diagnosis of cisplatin-associated AKI was supported by persistent decline in renal function and a concomitant drop in serum magnesium and potassium.

African American race and history of smoking were found to be major risk factors for development of cisplatin-induced AKI. Shord et al. [8] reviewed medical records of 62 patients receiving high dose cisplatin for head and neck cancer and reported that African American race was a risk factor for development of nephrotoxicity. It is unclear if it is due to genetic differences in drug metabolism or inherent predisposition to kidney disease. History of smoking has also been reported a risk factor for cisplatin associated nephrotoxicity [9]. It is possible that since we had a high proportion of American Americans (30%) and smokers (80%) in our study that these were noted to be risk factors. Although hypertension and diabetes has been noted by others [6] to be risk factors for cisplatin-associated AKI; our results suggests a trend but did not reach statistical significance, perhaps due to small numbers. There have been other reports where diabetes and hypertension have not been shown to affect cisplatin-induced nephrotoxicity [6,8,10]. Ours is the first report suggesting that placement of a PEG tube reduces the risk of AKI, perhaps by facilitating nutrition and hydration. Mizuno et al. [6] reported that patients with advanced cancer were at higher risk of cisplatin-associated AKI due to poor nutrition and hydration. Patients with advanced cancer had lower serum albumin, reflecting poor nutritional status. Recently, Kidera et al. [11] reported that magnesium supplementation also confers renal protection during cisplatin administration. PEG tube placement should be considered in patients with advanced cancer and with risk factors for AKI to maintain hydration, nutrition, and magnesium supplementation.

Our study shows that cisplatin infusion is associated with an acute drop in eGFR in all, regardless of clinical diagnosis of AKI. The reduction in eGFR on all patients suggests an acute vasoconstrictive effect of cisplatin on the kidney, similar to that seen with use of other nephrotoxic agents such as aminoglycosides, amphotericin B, etc. At one month after the infusion, eGFR improves in all patients but never returns to baseline and remains statistically significantly lower in those with higher stages of AKI. It is well known in general population that reduced renal function is associated with increased all-cause mortality. We found that in patients with higher stages of cisplatin-associated AKI also have inferior survivals. Unlike the general population, the decreased survival noted in the patients that developed severe AKI is due to early recurrence of cancer, or development of a cancer elsewhere, but not due to cardiovascular disease. Uremia has been reported to increase recurrence of squamous cell cancer but we do not have sufficient data to demonstrate such an association. Thus, cessation of cisplatin based chemotherapy due to its nephrotoxicity is a serious limitation to long-term control of cancer and calls for early adoption of renoprotective measures such as placement of PEG tube to maintain hydration, nutritional and electrolyte balance.

The limitations of our study are its retrospective nature and small sample size due to inclusion of patients with head and neck cancer only. The high incidence of AKI in our cohort could be due to the high risk nature of the group of patients receiving high dose cisplatin and concomitant radiation therapy to oral/pharyngeal cavity, making it difficult to maintain hydration and nutrition. Patients with cisplatin-associated AKI were noted to have decreased survival; it is unclear if this was due to abbreviated treatment or use of alternative agents with inferior oncologic effects. Currently, we do not have complete information on cancer treatment of individuals who developed AKI, and we had to discontinue cisplatin based therapy. Lastly, we do not have urinalysis on all patients after treatment so cannot comment on development of proteinuria in patients with CKD at follow-up. The strength of our study was the strict definition of AKI, ruling any other contributing factors and demonstration of concomitant decline in potassium and magnesium to support that the AKI was indeed related to cisplatin use. All patients were well hydrated and were given mannitol to maintain good urine flow and were monitored closely at days 3 and 7 for additional hydration and correcting electrolyte imbalance. Our study population was also heterogeneous with a third of patients being African Americans.

In conclusion, we report that over half the patients receiving high dose of cisplatin and radiation therapy for treatment of head and neck cancer develop AKI and 20% develop Stage III AKI, resulting in long-term loss of renal function and poor patient survival due to early cancer recurrence or development of secondary malignancy elsewhere. African American race and history of smoking were noted to be risk factors for development of AKI. PEG tube placement should be recommended to maintain hydration and nutrition, especially in high risk patients.
